# Knowledge, attitude, and practice toward postpartum depression among the pregnant and lying-in women

**DOI:** 10.1186/s12884-023-06081-8

**Published:** 2023-10-30

**Authors:** Kai Wang, Rui Li, Qingqing Li, Zhenzhen Li, Ning Li, Yandong Yang, Jia Wang

**Affiliations:** 1 Shanghai Yangzhi Rehabilitation Hospital (Shanghai Sunshine Rehabilitation Center), School of Medicine, Tongji University, Shanghai, China; 2 Department of Obstetrics and Gynecology, Binzhou Medical University Hospital, Binzhou, China; 3 Department of Obstetrics and Gynecology, Shanghai Putuo District Liqun Hospital, Shanghai, China

**Keywords:** Attitudes, Knowledge, Postpartum depression, Pregnant, Lying-in women

## Abstract

**Background:**

Postpartum depression (PPD) is considered an important public health problem, and early recognition of PPD in pregnant and lactating women is critical. This study investigated the knowledge, attitude, and practice (KAP) toward PPD among pregnant and lying-in women.

**Methods:**

This cross-sectional study was conducted at Binzhou Medical University Hospital between September 2022 and November 2022 and included pregnant and lying-in women as study participants. A questionnaire was designed by the researchers that included demographic data and knowledge, attitude, and practice dimensions. Correlations between knowledge, attitude, and practice scores were evaluated by Pearson correlation analysis. Factors associated with practice scores were identified by multivariable logistic regression.

**Results:**

All participants scored 6.27 ± 2.45, 36.37 ± 4.16, and 38.54 ± 7.93 93 from three sub-dimensions of knowledge, attitudes, and practices regarding PPD, respectively, with statistical differences in the three scores by age, education, and job status (p < 0.05). There were no significant differences between maternal (6.24 ± 2.34, 36.67 ± 3.82 and 38.31 ± 7.27, respectively) and pregnant women (6.30 ± 2.49, 36.00 ± 4.53 and 38.83 ± 8.69, respectively) in the total scores of knowledge, attitude, and practice dimensions. According to the results of multivariate logistic regression, the knowledge (OR = 1.235[1.128–1.353], P < 0.001) and attitude (OR = 1.052[1.005–1.102], P = 0.030) dimension scores were factors influencing the practice dimension scores.

**Conclusion:**

The KAP of pregnant and lying-in women toward PPD is low. This study suggests that maternal awareness of PPD should be increased through the knowledge and attitudinal dimensions. Preventing PPD in pregnant and lying-in women can be achieved by improving both dimensions, thus enhancing practice.

**Supplementary Information:**

The online version contains supplementary material available at 10.1186/s12884-023-06081-8.

## Background

Pregnancy is a complex process that can lead to dramatic changes in female’s physical, psychological, and social roles. Since pregnancy and birth-giving are both major life events and traumatic processes, postpartum is often considered to be the most risky stage for women to develop depression [1, 2]. Postpartum depression (PPD) is a cross-disciplinary disorder between obstetrics and psychology, which not only has a negative impact on the health of a lying-in woman and her marriage and family but also on breastfeeding, the mother-infant relationship, and the growth& development and emotional behavior of the infant. In more serious cases, infanticide and suicidal tendencies or behaviors may even occur, causing great harm to the mother’s family and society [3, 4].

Risk factors for PPD have been discussed more in previous studies. Pregnant women’s psychophysiological disorders can influence PPD. A meta-analysis by Liu et al. [5] concluded that PPD is relatively higher in developing countries and that gestational diabetes and a history of depression were considered risk factors. In addition, the influence of family on postnatal depression is equally essential. Xie et al. [6] concluded that postnatal family support, especially from the husband, is an essential protective factor for postnatal depression. Poreddi et al. [7] investigated family members in terms of both knowledge and attitudes and concluded that there is an urgent need to address misconceptions and negative stereotypes about postnatal depression among family members. Since there is still a COVID-19 pandemic and the prevalence and odds of PPD are significantly higher [8], there is a need for greater awareness and guidance on PPD.

A knowledge, attitude, and practice (KAP) survey is a structured survey method that can be used to investigate the current state of specific people (i.e., pregnant and lying-in women) toward a specific subject (i.e., PPD) [9]. The practice represents the various behaviors and actions taken by the surveyed people toward the specific subject. Previous studies examined the KAP toward related subjects in different populations. Temtanakitpaisan et al. [10] investigated pregnant women’s awareness of pelvic floor muscle training (PFMT) using a KAP questionnaire. A questionnaire revealed that women perceive that PFMT impacts their mental health and quality of life positively. Aiga et al. [11] used a KAP questionnaire to assess the effectiveness of maternal and child health manuals to intervene in maternal behavior change. Using this questionnaire allowed for greater effectiveness of maternal and child health manuals. KAP surveys are becoming increasingly important as they can enhance the views and perceptions of specific people about certain things. No data are available about the maternal awareness of PPD through the three KAP dimensions. In our study, a high-reliability and validity questionnaire was designed to investigate the three dimensions of maternal awareness. Furthermore, the practice dimension was used to guide maternal awareness to reduce the occurrence of PPD.

## Methods

### Study design and participants

This cross-sectional study was conducted between September 2022 and November 2022 at Binzhou Medical University Hospital and included pregnant (any gestational age) and lying-in women who volunteered to participate in this study as participants. Pregnant women were defined as reproductive-age women with a history of sexual activity, amenorrhea or menstrual abnormalities, positive blood or urine hCG test indicating pregnancy, and ultrasound findings of intrauterine gestational sac or embryo. The lying-in women were those with postpartum hospitalization or attending follow-up visits within 42 days after delivery. This study received ethical approval from the Ethics Committee of Binzhou Medical University Hospital (approval no. LW-37), and informed consent was obtained from the participants.

### Procedures

Based on Expert Consensus on Guidelines for the Management of Postpartum Depression and previously published studies [12–14], the KAP questionnaire was self-designed and included four dimensions: (1) demographic data of the participants; (2) knowledge dimension, consisting of 10 questions (items K4 and K8 were incorrect statements), scored 1 point for correct answers and 0 points for wrong or unclear answers, with total scores ranging from 0 to 10 points; (3) attitude dimension, containing 10 questions scored using a 5-point Likert scale ranging from very positive (5 points) to very negative (1 point), with total scores ranging from 10 to 50 points; (4) practice dimension, including 11 items (item P1 included seven subitems) scored using 5-point Likert scale ranging from always (5 points) to never (1 point), with total scores ranging from 11 to 55 points. Higher scores indicated better KAP.

A small pre-test (50 copies) was conducted before the formal launch, and Cronbach’s α was 0.9266, suggesting a high internal consistency. The results of the confirmatory factor analysis are now shown in Supplementary Figure S1 ((CFI = 0.817 (> 0.800 is good); IFI = 0.818 (> 0.800 is good); RMSEA = 0.069 (< 0.080 is good); CMIN/DF = 3.859 (> 1; 1–3 is excellent, 3–5 is good)), indicating that the questionnaire has good reliability.

Online e-questionnaires are created through the Wen-Juan-Xing online platform in China (https://www.wjx.cn/app/survey.aspx), the questionnaires were distributed, and the data were collected from anonymous participants through Moments forwarding and WeChat group promotion. In order to ensure the quality and completeness of the questionnaire results, each IP address can only be used once for submission. Each question was compulsory. All questionnaires were checked for completeness, consistency, and validity by members of the research team.

### Statistical analysis

Stata 17.0 (Stata Corporation, College Station, TX, USA) was used for statistical analysis. The Kolmogorov-Smirnov test was used to examine the distribution of the continuous variables, and most of them were not conforming to a normal distribution. They were presented as median (P25, P75) and analyzed using the Wilcoxon test. Age was presented as means ± standard deviations (SD). Categorical data were expressed as n (%). Pearson’s correlation was used to analyze the correlation between knowledge scores, attitude scores, and practice scores. Univariable and multivariable logistic regression analyses were used to analyze the factors influencing practice. Sufficient knowledge, positive attitude, and proactive practice were defined as scores exceeding 70% of the total score for each respective dimension [15]. All statistical tests were performed using two-sided tests, and P < 0.05 were considered statistically significant.

In the absence of relevant literature on pelvic floor dysfunction and pelvic floor ultrasound in our population, the sample size for the study was calculated with an anticipated proportion of mask-wearing practice as 50%, at a 95% confidence level and 5% error margin the required sample size of 384 was calculated. Considering a 70% response rate, at least 549 participants were needed to be included [16].

## Results

### Baseline and KAP scores in pregnant and lying-in women

Five hundred ninety-four pregnant women completed the questionnaire, with most participants aged 25–35 years (78.5%), nearly two-thirds of whom were urban residents, and more than two-thirds had a bachelor’s degree or higher. The total score of the knowledge dimension for all participants was 6.27 ± 2.45, with statistical differences (p < 0.05) for different age, places of residence, education, work status, and para; the total score of the attitude dimension was 36.37 ± 4.16, with statistical differences for different age, residence, education, work status, gravida, para, and current gestational age (p < 0.05), the total score of the practice dimension was 38.54 ± 7.93, and there were statistical differences in the scores of different age, education, and work status (p < 0.05) (Table 1).

**Table 1:** Baseline characteristics and KAP scores

Variables	N (%)	Knowledge score		Attitude score		Practice score	
		Mean ± SD	P	Mean ± SD	P	Mean ± SD	P
**Total**		6.27 ± 2.45		36.37 ± 4.16		38.54 ± 7.93	
**Age**			0.014		0.003		0.012
≤ 24	62(10.44)	6(3.75-8)		36(32-37.25)		35(29.75-40)	
25–30	260(43.77)	7(6–8)		37(34–40)		39(34-45.75)	
31–35	206(34.68)	7(5–8)		37(34–39)		39(33–44)	
≥ 36	66(11.11)	7(5–8)		36(32–38)		38(33.75-43)	
**Residence**			<0.001		<0.001		0.261
Non urban	224(37.71)	6(4–8)		35(32.25-38)		38(32.25-44)	
Urban	370(62.29)	7(6–8)		37(35–40)		39(33.75-44)	
**Education**			<0.001		<0.001		0.002
Middle School/Below	98(16.50)	6(3–7)		34(32–37)		37(31–42)	
High School/Technical secondary school	86(14.48)	6(4–8)		35(32–37)		37(31.75-42)	
Junior college/Bachelor’s degree	378(63.64)	7(6–8)		37(35–40)		39(34–45)	
Master’s degree/Above	32(5.39)	8(7–9)		39.5(36–42)		42(37–45)	
**Work Status**			<0.001		<0.001		0.003
Employed	359(60.44)	7(6–8)		37(35–40)		39(34–45)	
Unemployed	27(4.55)	5(4–6)		37(34–39)		36(32–41)	
Self-employed	49(8.25)	6(4.5–7.5)		35(30–38)		37(31–43)	
Housewife	99(16.67)	6(4–8)		35(33–37)		39(33–43)	
Others	60(10.10)	7(4–8)		36(32.25-38)		36(29.25-40)	
**Income**			0.287		0.285		0.321
<5000	274(46.13)	7(5–8)		36(34–39)		38(33–44)	
5000–10,000	236(39.73)	7(6–8)		37(34-39.75)		38(33–44)	
>10,000	84(14.14)	7(6–8)		37(35–39)		40(35.25–44.75)	
**Marital status**							
Married	594(100.00)						
**Gravida**			0.248		0.004		0.594
1	260(43.77)	7(5–8)		37(34–40)		39(33–45)	
2	168(28.28)	7(5.25-8)		36(34-38.75)		37(32.25–43.75)	
3	87(14.65)	7(5–8)		36(33–39)		39(34–43)	
≥ 4	79(13.30)	7(4–8)		35(33–38)		39(33–44)	
**Para**			0.028		<0.001		0.896
0	120(20.20)	7(6–8)		37.5(35.25-40)		39(33-44.75)	
1	266(44.78)	7(5–8)		37(34-39.25)		38(33–44)	
2	148(24.92)	7(5–8)		36(33–38)		38(33–44)	
≥ 3	60(10.10)	6(4–8)		35(32-37.75)		39(34–43)	
**Current stage**			0.548		0.073		0.216
Pregnancy period	330(55.56)	7(5–8)		37(34–39)		38(33–43)	
The first trimester of pregnancy (0–12 weeks)	3(0.51)						
The second trimester of pregnancy (13–27 weeks)	11(1.85)						
The third trimester of pregnancy (from 28 weeks)	306(51.52)						
No answer	10(1.68)						
postpartum period	264(44.44)	7(5–8)		36(33–39)		39(33.25-45)	
Postnatal hospitalisation	149(25.08)						
Review at 42 days postpartum	42(7.07)						
Review later than 42 days postpartum	60(10.10)						
No answer	13(2.19)						
**Medical insurance type**							
Social medical insurance	586(98.65)	7(5–8)	0.473	36(34–39)	0.793	38.5(33–44)	0.898
Commercial insurance	42(7.07)	7(5–8)	0.938	36.5(34-39.25)	0.587	40.5(36-48.25)	0.021
No insurance	6(1.01)	5(1.5-9)	0.784	36(35.25–40.75)	0.656	36(29.5-45.75)	0.625

### Knowledge, attitude, and practice scores

The total scores for the three dimensions of knowledge, attitude, and practice were 6.24 ± 2.43, 36.67 ± 3.82 and 38.31 ± 7.27 for pregnant women and 6.30 ± 2.49, 36.00 ± 4.53 and 38.83 ± 8.69 for lying-in women, respectively, and there was no statistical difference between the total scores of pregnant women and mothers in the three dimensions (P > 0.05). However, on the attitude dimension, pregnant women had scores of 4.16 ± 1.13 for item A1, 4.41 ± 0.78 for A2, and 2.75 ± 1.05 for A6, and lying-in women had scores of 3.75 ± 1.36, 4.22 ± 0.98 and 2.94 ± 1.09 for items A1, A2, and A6, respectively, with statistical differences in the scores of the three subscales (p < 0.001; 0.014; 0.038) The remaining detailed scores are shown in Table 2. In terms of knowledge dimension, 363 (61.11%) participants were correct that PPD is a common puerperal psychiatric syndrome that occurs within four weeks, 475 (79.97%) participants were correct that the occurrence of PPD is related to genetic, neuroendocrine disorders and psychosocial factors, 522 (87.88%) participants believed that PPD needs to be treated, and 417 (70.20%) participants were correct that medication should be avoided during breastfeeding, and overall, the majority of participants had a relatively correct understanding of PPD (Table 3). For Supplementary Table 1, the percentages of each sub-item score in the attitude dimension; for Supplementary Table 2, the percentages of each item score in the practice dimension.

**Table 2:** Group comparison

Factor or Statement	Participants		p
	Pregnant Women	Lying-in woman	
Knowledge	7(5–8)	7(5–8)	0.548
Attitude			
Total	37(34–39)	36(33–39)	0.073
A1. Postpartum depression is fault-finding or hypocritical and a sign of emotional weakness	4(4–5)	4(3–5)	<0.001
A2. Having postpartum depression is not a big deal	5(4–5)	4(4–5)	0.046
A5. Worrying about the upbringing of your baby	3(2–3)	3(2–4)	0.171
A6. Worrying about your original life path	3(2–4)	3(2–4)	0.032
A8. Life will get back on track	4(4–5)	4(4–5)	0.815
Practice			
Total	38(33–43)	39(33.25-45)	0.216
Specific behaviors (items P1.1-P1.7)	26(23–29)	26(22–30)	0.743
P4. Be proactive in getting screened for postpartum depression	2(2–3)	3(2–4)	0.025
P7. Encouraging family and friends to receive treatment	5(4–5)	5(3–5)	0.425

**Table 3:** Knowledge Dimension

Knowledge	N (%)	
	Correct	Wrong/Unclear
K1. Postpartum depression is a common puerperal psychiatric syndrome that occurs within 4 weeks after birth.	363(61.11)	231(38.89)
K2. The occurrence of postpartum depression is related to genetic factors, neuroendocrine dysfunction, neurobiochemical factors, and psychosocial factors.	475(79.97)	119(20.03)
K3. Symptoms of postpartum depression include depressed mood, diminished interest, insufficient energy, and even suicidal or infanticidal tendencies in severe cases.	497(83.67)	97(16.33)
K4. No treatment is needed for postpartum depression. (Wrong)	522(87.88)	72(12.12)
K5. Postpartum depression is the result of dramatic postpartum changes of hormonal and life in a short time and has little to do with the temper of birth-giving women.	268(45.12)	326(54.88)
K6. More than 80% of postpartum depression is mild, and if supplemented with active psychological counseling or treatment, it can be self-relieving within 1 to 6 months and generally does not affect normal life too much.	211(35.52)	383(64.48)
K7. Estrogen levels of birth-giving women drop rapidly after childbirth, causing changes in mood and behavior, so poor mood after birth-giving is not something they can fully control.	455(76.60)	139(23.40)
K8. Brief, mild symptoms of depression also need to be taken very seriously. (Wrong)	11(1.85)	583(98.15)
K9. The most immediate effect of postpartum depression is a reduction in breast milk production.	332(55.89)	262(44.11)
K10. All psychotropic drugs will leach into breast milk, and the long-term developmental effects of infant exposure through breast milk are not known, so as a matter of principle, medication needs to be avoided during breastfeeding as much as possible	417(70.20)	117(19.70)

### Correlation analysis between the scores of the three different dimensions

By analyzing the two-by-two Pearson coefficients between the total scores of the three dimensions, the results showed that the Pearson coefficient between the practice score and attitude score was 0.162 (p < 0.001), the Pearson coefficient between practice score and knowledge score was 0.318 (p < 0.001), the Pearson coefficient between attitude score and knowledge score was 0.165 (p < 0.001), and all dimensions were positively correlated with each other (Table 4).

**Table 4:** Pearson correlation analysis

	Knowledge	Attitude	Behavior
Knowledge	1		
Attitude	0.166*	1	
Practice	0.306*	0.177*	1

*P < 0.001

### Factors associated with the practice scores

Univariate logistic regression analysis revealed that knowledge dimension scores, attitude dimension scores, age, education, work status, and current stage were the factors that influenced the practice dimension scores. When incorporating these factors into a multivariate logistic regression model, the results showed that knowledge scores (OR = 1.235, 95%CI: 1.128–1.353, P < 0.001 per 1 point), attitude scores (OR = 1.052, 95%CI: 1.005–1.102, P = 0.030 per 1 point), work status‘s others (OR = 0.431, 95%CI: 0.193–0.961, P = 0.040 vs. employed) and Current stage‘s postpartum period (OR = 1.509, 95%CI: 1.043–2.182, P = 0.029 vs. pregnancy period) were significantly associated with a higher practice score (Table 5).

**Table 5:** Univariate logistic regression analysis

Factors	Univariate logistic regression		Multivariate logistic regression	
	OR (95%CI)	P	OR (95%CI)	P
**Knowledge Score**	1.263(1.159–1.377)	<0.001	1.235(1.128–1.353)	<0.001
**Attitude Score**	1.069(1.024–1.115)	0.002	1.052(1.005–1.102)	0.030
**Age**				
≤ 24	ref		ref	
25–30	2.099(1.083–4.069)	0.028	1.306(0.629–2.714)	0.474
31–35	1.857(0.944–3.655)	0.073	1.174(0.563–2.448)	0.670
≥ 36	1.759(0.789–3.920)	0.167	1.118(0.468–2.672)	0.801
**Residence**				
Non urban	ref			
Urban	0.985(0.692–1.402)	0.933		
**Education**				
Middle School/Below	ref		ref	
High School/Technical secondary school	0.952(0492-1.842)	0.884	0.917(0.450–1.871)	0.812
Junior college/Bachelor’s degree	1.452(0.884–2.384)	0.141	1.009(0.524–1.942)	0.979
Master’s degree/Above	3.138(1.373–7.173)	0.007	1.907(0.719–5.052)	0.194
**Work Status**				
Employed	ref		ref	
Unemployed	3.256(1.553–6.827)	0.002	0.749(0.278–2.016)	0.567
Self-employed	1.619(0.512–5.120)	0.412	1.276(0.628–2.592)	0.501
Housewife	2.747(1.088–6.940)	0.033	1.287(0.709–2.338)	0.407
Others	2.833(1.245–6.450)	0.013	0.431(0.193–0.961)	0.040
**Income**				
<5000	ref			
5000–10,000	1.079(0.744–1.565)	0.688		
>10,000	1.279(0.767–2.132)	0.346		
**Gravida**				
1	ref			
2	0.706(0.464–1.075)	0.104		
3	0.883(0.529–1.474)	0.634		
≥ 4	0.771(0.448–1.325)	0.346		
**Para**				
0	ref			
1	0.769(0.490–1.208)	0.254		
2	0.776(0.467–1.287)	0.325		
≥ 3	0.772(0.400-1.491)	0.441		
**Current stage**				
Pregnancy period	ref		ref	
postpartum period	1.462(1.037-0.064)	0.030	1.509(1.043–2.182)	0.029

## Discussion

This preliminary cross-sectional study explores pregnant and lying-in women’s perceptions of PPD. We surveyed participants in three dimensions by creating a KAP questionnaire. The study results showed that maternal scores in the three dimensions differed across states, pregnant and maternal women had good knowledge of PPD in more than half of the participants, and that practice dimension scores correlated with knowledge and attitude scores. However, disregard for PPD remained prevalent among a minority of participants. Therefore, the KAP survey allows for a preliminary understanding of the different dimensions of PPD in the maternal population. Based on the results, education and coping measures for PPD can be developed and implemented to address these biases and negative stereotypes associated with PPD.

Studies on the link between KAP and PPD are rare, especially during the COVID-19 pandemic in recent years, which may have had a direct psychological and social impact on pregnant women and women who have just given birth due to the relative restriction of social activities. In this study, we found statistically significant differences in their scores on the three dimensions of KAP across age groups, education, and work status. This is more consistent with the findings of previous studies. Silva et al. [17] found statistical differences in anxiety-depression scores by maternal age through a questionnaire survey of pregnant women. At the same time, PPD was associated with age and a high rate of PPD among lying-in women, which is associated with maternal anxiety and should further enhance mental health during pregnancy and puerperium. A meta-analysis of a Chinese study by Nisar et al. [18] found that the prevalence of perinatal depression showed an increasing trend in the last decade. At the same time, a higher educational level was a protective factor. Our study showed that as educational background increased, higher education was associated with higher KAP scores. Using a sample analysis of 15,000 mothers in a nationwide French cohort, Nakamura et al. [19] found occupational rank. Employment and higher socioeconomic status made possible by education reduced the occurrence of PPD, with higher scores for those who were employed than for those who were not employed and those who worked part-time in the three dimensions. Overall, we should pay further attention to pregnant women and lying-in women with low scores on the three dimensions.

Although our results showed no statistically significant differences in the scores of women during pregnancy and lying-in on the three dimensions, there were differences in the scores obtained by women at different periods on some subitems. Analysis of the subitems with differences showed that women during pregnancy took PPD more seriously in their mindset and actions and were more willing to be proactive in being screened for PPD. A woman in labor is likely to worry about how to care for her child, which can cause them anxiety. Also, the hormonal changes and frequent mood swings from pregnant women to mothers may affect their perception of PPD. Pięta et al. [20] argue that special attention should be paid to women who experience negative mental states during pregnancy. They argue that mood changes during pregnancy, labor, and lying in are often extreme and can change significantly in short periods. For their part, researchers such as Andrzej [21] suggest practical actions in the field of public health, which would raise awareness among young women and pregnant women and make them aware of the importance of physical activity for their pregnancy and childbirth process. We also believe we should provide psychological support to pregnant women to enhance their awareness of PPD.

Our results show a positive correlation between maternal scores on the three dimensions, with the three dimensions influencing each other. Previous studies generally agree that there is a correlation between knowledge, attitudes, and practices. Alsabi [22] surveyed social support networks during the COVID-19 pandemic in Malaysia, and they found that public perceptions of PPD were correlated with their knowledge and attitudes. Highet et al. [23] investigated three aspects of perinatal depression awareness, attitudes, and knowledge among Australians, and they concluded that people without mental health training had lower awareness and attitudes towards the etiology of PPD, and when awareness increased, the attitudes towards PPD also changed. Jones et al. [24] conducted a national survey of midwives and found that when midwives are professionally educated, and their knowledge is improved, it also leads to better cognitive and practical skills in assessing and caring for women with PPD. Likewise, Elshatarat [25] believes that educating healthcare professionals and improving their understanding of PPD will improve their knowledge, practice, and self-confidence. We also believe daily information should be provided to pregnant women to understand PPD and prevent it by increasing knowledge and positive attitudes towards PPD.

For maternal practice aspects, we concluded through multivariate logistic regression analysis that the knowledge and attitude dimensions influence practice. At the same time, the postpartum period will have a higher practice score than the pregnancy period (OR = 1.509 [1.043–2.182]). It is essential for maternity to put into practice both psychological and behavioral aspects of the intervention. Earls et al. [26] argued that payment should be advocated for increased training in the screening and treatment of PPD, with significant support for the mother-infant relationship, and for the identification of perinatal depression to be incorporated into pediatric practice. A randomized controlled trial by Meng et al. [27] concluded that a circulatory care intervention positively affected postpartum anxiety and depression and significantly reduced the incidence of PPD. We believe that pregnant and lying-in women should be motivated to act and seek interventions to prevent PPD. A meta-analysis by Zhou et al. [28] also concluded that pregnant women should actively use portable electronic devices to address depressive symptoms in postpartum women, increase knowledge of PPD through electronic devices to change their attitudes and use mHealth interventions to complement routine clinical care for PPD. Therefore, maternal practice is crucial to reducing the incidence of PPD by providing maternal guidance through knowledge and attitudes so that they can be more proactive in seeking professional help. It may be that hormonal changes and the impact of the newborn and the family on the mother make postpartum women more aggressive in seeking help. However, better results are obtained with behavioral interventions early in pregnancy.

This study has some limitations; firstly, this sample is from a single center. At the same time, there is also an insufficient sample size; future multi-center studies are needed to expand the sample size. Secondly, despite the high Cronbach’s alpha coefficient, the KAP survey may need to be validated by more institutions as it is artificially designed.

### Conclusions and recommendations

This KAP study revealed that pregnant and lying-in women had relatively good knowledge and positive attitudes toward PPD. Better knowledge and positive attitudes can also improve practice toward PPD. Therefore, it is essential to develop appropriate policies to educate mothers about PPD and improve their attitudes and practices toward PPD. Such improvements could have public health implications, considering that PPD is associated with serious morbidity.

### Electronic supplementary material

Below is the link to the electronic supplementary material.

**Supplementary Material 1: Supplementary Table 1**. The score distribution of “attitude” dimension

**Supplementary Material 2: Supplementary Table 2**. The score distribution of “practice” dimension

**Supplementary Material 3: Supplementary Figure S1**. Results of the confirmatory factor analysis

## Data Availability

All data generated or analyzed during this study are included in this published article [and its supplementary information files].

## List of abbreviations

KAP: Knowledge, Attitude and Practice
PPD: Postpartum depression
SD: Standard deviations
