# Supplementary material for: Knowledge, attitude, and practice toward postpartum depression among the pregnant and lying-in women
Source: BMC Pregnancy Childbirth. 2023 Oct 30;23:762. doi: 10.1186/s12884-023-06081-8 (PMC10614410; doi:10.1186/s12884-023-06081-8)
Supplement: Supplementary file 1 — Supplementary Material 1: Supplementary Table 1. The score distribution of “attitude” dimension [file 12884_2023_6081_MOESM1_ESM.docx]

**Supplementary Table 1** The score distribution of “attitude” dimension.

|  | Scored 1 point | Scored 2 points | Scored 3 points | Scored 4 points | Scored 5 points |
| --- | --- | --- | --- | --- | --- |
| A1 | 8.42% | 7.24% | 7.07% | 32.83% | 44.44% |
| A2 | 2.69% | 2.19% | 5.05% | 40.07% | 50.00% |
| A3 | 38.38% | 34.51% | 8.08% | 13.13% | 5.89% |
| A4 | 2.69% | 2.36% | 14.14% | 60.27% | 20.54% |
| A5 | 11.78% | 30.30% | 33.67% | 19.87% | 4.38% |
| A6 | 10.94% | 28.62% | 32.32% | 22.39% | 5.72% |
| A7 | 2.69% | 9.09% | 21.72% | 50.17% | 16.33% |
| A8 | 2.19% | 4.21% | 14.65% | 50.00% | 28.96% |
| A9 | 2.02% | 0.51% | 7.58% | 47.81% | 42.09% |
| A10 | 1.68% | 0.34% | 4.38% | 37.04% | 56.57% |
