# Supplementary material for: Knowledge, attitude, and practice toward postpartum depression among the pregnant and lying-in women
Source: BMC Pregnancy Childbirth. 2023 Oct 30;23:762. doi: 10.1186/s12884-023-06081-8 (PMC10614410; doi:10.1186/s12884-023-06081-8)
Supplement: Supplementary file 2 — Supplementary Material 2: Supplementary Table 2. The score distribution of “practice” dimension [file 12884_2023_6081_MOESM2_ESM.docx]

**Supplementary Table 2** The score distribution of “practice” dimension.

|  | Scored 1 point | Scored 2 points | Scored 3 points | Scored 4 points | Scored 5 points |
| --- | --- | --- | --- | --- | --- |
| P1.1 | 11.95% | 8.92% | 48.32% | 17.00% | 13.80% |
| P1.2 | 11.45% | 8.59% | 48.15% | 17.00% | 14.81% |
| P1.3 | 2.36% | 2.36% | 20.03% | 33.00% | 42.26% |
| P1.4 | 3.20% | 5.89% | 34.68% | 31.14% | 25.08% |
| P1.5 | 3.03% | 4.88% | 26.60% | 32.15% | 33.33% |
| P1.6 | 2.69% | 6.06% | 27.27% | 33.00% | 30.98% |
| P1.7 | 1.68% | 1.52% | 16.67% | 37.04% | 43.10% |
| P4 | 22.73% | 30.30% | 23.06% | 11.45% | 12.46% |
| P5 | 15.66% | 17.00% | 20.20% | 20.54% | 26.60% |
| P6 | 38.89% | 13.47% | 18.01% | 12.46% | 17.17% |
| P7 | 4.04% | 7.24% | 12.63% | 20.71% | 55.39% |
