# Supplementary figures and images for: Knowledge, attitude, and practice toward postpartum depression among the pregnant and lying-in women
Source: BMC Pregnancy Childbirth. 2023 Oct 30;23:762. doi: 10.1186/s12884-023-06081-8 (PMC10614410; doi:10.1186/s12884-023-06081-8)

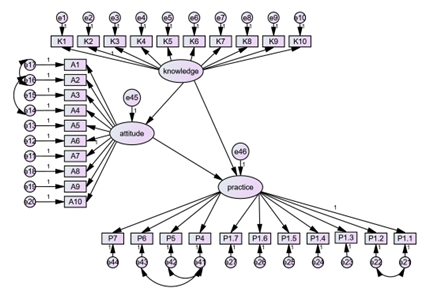


**Supplementary Figure S1.** Results of the confirmatory factor analysis.

Supplement: Supplementary file 3 — Supplementary Material 3: Supplementary Figure S1. Results of the confirmatory factor analysis [file 12884_2023_6081_MOESM3_ESM.docx]
